# Denosumab can be used successfully as a bridge to surgery in patients with severe hypercalcemia due to primary hyperparathyroidism

**DOI:** 10.20945/2359-3997000000408

**Published:** 2021-09-29

**Authors:** Mohammad Shafi Kuchay, Anu Mathew, Parjeet Kaur, Sunil Kumar Mishra

**Affiliations:** 1 The Medicity Hospital Division of Endocrinology and Diabetes, Medanta Gurugram Haryana India Division of Endocrinology and Diabetes, Medanta – The Medicity Hospital, Gurugram, Haryana, India

## Abstract

Severe hypercalcemia is a medical emergency that requires immediate and aggressive management. Primary hyperparathyroidism (PHPT) often causes severe hypercalcemia. Volume resuscitation, parenteral salmon calcitonin, and administration of intravenous bisphosphonates are common measures used to stabilize patients. However, the use of these measures is inadequate in several patients and may even be contraindicated in individuals with renal insufficiency or severe systemic illness. This study demonstrated the efficacy and safety of denosumab in patients with severe hypercalcemia due to PHPT, when immediate surgery was not feasible. We present four patients with severe hypercalcemia due to PHPT. Immediate surgery was not feasible because the patients had severe systemic illness, such as seizures and altered sensorium (case 1); acute severe pancreatitis (cases 2 and 3); or coronavirus disease 2019 pneumonia (case 4). Intravenous normal saline and parenteral salmon calcitonin were inadequate for controlling hypercalcemia. Intravenous bisphosphonates were avoided because of severe systemic illness in all cases and impaired renal function in three cases. Denosumab was administered to control hypercalcemia and allow the stabilization of patients for definitive surgical management. Following denosumab administration, serum calcium levels normalized, and general condition improved in all patients. Three patients underwent parathyroidectomy after two weeks and another patient after eight weeks. The use of denosumab for the management of severe hypercalcemia due to PHPT is efficacious and safe in patients when immediate surgical management is not feasible due to severe systemic illness.

## INTRODUCTION

Denosumab is a human monoclonal antibody that binds and inactivates receptor activator of nuclear factor kappa-B ligand (RANKL), thus suppressing osteoclast-induced bone resorption. It is used for the treatment of hypercalcemia mainly associated with malignancy in the setting of renal impairment and also in cases of refractoriness to bisphosphonates ( 1 ).

Primary hyperparathyroidism (PHPT), a mostly benign condition caused by parathyroid adenoma, can present with life-threatening hypercalcemia, defined as a serum total calcium level > 14 mg/dL. Bisphosphonates are widely used for the treatment of hypercalcemia along with saline diuresis and parenteral salmon calcitonin and calcimimetics, such as cinacalcet ( 1 ). As bisphosphonates have the potential to cause nephrotoxicity, their use in patients with severe hypercalcemia and renal impairment is usually avoided. Hence, denosumab, which is excreted by the reticuloendothelial system, is being tested as a bridge therapy until the patient is stable for parathyroidectomy. The DENOCINA trial demonstrated the efficacy and safety of denosumab and cinacalcet for the management of PHPT, as it effectively reduced calcium levels and preserved bone density ( 2 ). In this report, we describe four cases in which severe hypercalcemia secondary to PHPT was managed with intravenous denosumab as immediate surgery was not feasible due to severe systemic illness.

## CASE REPORTS

All patients were managed in our hospital, and informed consent was obtained from all participants.

### Patient 1:

A 75-year-old female with hypertension for the past 20 years presented to the emergency department with sudden-onset giddiness and a fall in the bathroom followed by altered sensorium. She had a history of insidious onset lower back pain for two years, which worsened over the past six months, and she was unable to walk. She also complained of increased thirst and urine output for two months. In the emergency department, her vital signs were stable, and systemic examination was unremarkable except for drowsiness without any focal deficits. Laboratory workup revealed severe hypercalcemia (calcium level, 14.6 mg/dL) and elevated parathyroid hormone (PTH) levels ( Table 1 ). She was treated with intravenous fluids and injected with salmon calcitonin. Ultrasonography of the neck revealed a well-defined hypoechoic nodule (40 mm × 13 mm × 16 mm) in the left inferior parathyroid region, which was confirmed by a sestamibi parathyroid scan with SPECT images. In the hospital, she had a seizure followed by worsening of the sensorium and elevation of serum calcium levels (17.0 mg/dL). In view of her life-threatening condition, she underwent low-calcium hemodialysis. Denosumab (60 mg) was administered subcutaneously. Intravenous bisphosphonate was avoided, as the patient had acute kidney injury (AKI). Her calcium levels normalized gradually with no further requirement for hemodialysis. Subsequently, the patient underwent parathyroidectomy after stabilization. Histopathological examination revealed a single parathyroid adenoma. She developed hypocalcemia (serum total calcium, 7.6 mg/dL) on the third day of surgery, which was managed with oral calcium and vitamin D supplementation.

**Table 1:** Baseline patient characteristics

Variable	Case 1	Case 2	Case 3	Case 4	Normal range
Age (years)/Gender	75/F	35/F	56/F	47/M	–
Presenting condition	Altered sensorium	Acute pancreatitis	Acute pancreatitis	COVID-19 pneumonia	–
Total calcium, mg/dL	14.6	12.6	16.1	15.3	8.5-10.2
Serum albumin, g/L	4.2	4.2	3.5	3.9	3.5-5.0
Serum urea, mg/dL	92	38	58	48	15-36
Serum creatinine, mg/dL	1.9	1.0	1.6	1.8	0.7-1.2
Serum ALP, U/L	161	330	212	535	30-120
25OHD, ng/mL	22.0	<8.0	24.8	115.0	30-100 ng/mL
Intact-PTH (pg/mL)	1566	682	1251	2308	(15.0-68.3)
USG abdomen	Bilateral polycystic kidneys, no calculi	Bilateral renal concretions, Bulky pancreas	Bulky pancreas and renal calculi	Renal calculi	–
USG neck	Left inferior parathyroid lesion	Left inferior parathyroid lesion	Parathyroid lesion on the Posterolateral aspect of right lobe of thyroid	All four parathyroid glands were enlarged	–
Sestamibi parathyroid scan	Left inferior parathyroid lesion	Left inferior parathyroid lesion	Right superior parathyroid lesion	Two right parathyroid nodules	–
Intra-operative PTH decreased by	∼70%	>80%	>75	∼90%	>50%
Histopathology	Parathyroid adenoma	Parathyroid adenoma	Parathyroid adenoma	3 parathyroid adenomas; one gland hyperplasia	–

ALP: alkaline phosphatase; 25OHD: 25-hydroxyvitamin D; PTH: parathyroid hormone; USG: ultrasonography.

She had normal serum calcium levels two weeks after surgery ( Table 1 ).

### Patient 2:

A 35-year-old female with no previous comorbidities presented to the emergency department with a history of recurrent vomiting and severe abdominal pain for three days. Pain radiated to her back with no obstipation or constipation. The patient was diagnosed with severe acute pancreatitis. A blood test revealed hyperamylasemia (4,396 IU/L) and hyperlipasemia (7,674 IU/L). Serum total calcium (12.6 mg/dL) and PTH levels (682 pg/mL) were elevated ( Table 1 ). Abdominal computed tomography (CT) showed acute necrotizing pancreatitis with 70%-80% necrosis of the body and tail of the pancreas with partial splenic vein thrombosis. It also showed bilateral renal calculi. She was managed with intravenous fluids, antibiotics, and nil per oral. Injection of salmon calcitonin was also administered to treat hypercalcemia. Ultrasonography of the neck revealed a well-defined homogenous hypoechoic mass (19 mm × 16 mm × 13 mm) inferior to the left lower pole of the thyroid gland with peripheral and eccentric vascularity. This was further confirmed by a sestamibi parathyroid scan with SPECT images. As she had persistent hypercalcemia with underlying acute necrotizing pancreatitis, she was unfit for emergency parathyroidectomy. Hence, to control hypercalcemia, denosumab (60 mg) was administered subcutaneously. In view of acute pancreatitis with underlying systemic inflammatory response syndrome, the possibility of AKI was considered; therefore, intravenous bisphosphonate was avoided. After denosumab injection, her serum calcium level normalized within 36 h ( Table 2 ) and remained normal until the time of surgery, which was performed two weeks after initial injection. Her postoperative recovery was unremarkable. Histopathological examination revealed a single parathyroid adenoma.

**Table 2:** Serial serum total calcium concentrations before and following denosumab administration

Days	Admission	1	2	3	5	7	14
Case 1	14.1	13.5	17.1 #	14.0 *	12.3	10.7	10.2
Case 2	12.5	10.1 (M)	12.8 *	11.8 (M)	9.8	9.6	9.3
		12.5 (E)		10.9 (E)			
Case 3	16.1	14.3	12.7	13.5	15.0 (M) *	10.3 (M)	8.8
					13.0 (E)	10.1 (E)	
Case 4	15.3	15.1 *	14.9 (M)	12.1	10.6	9.7	10.1
			12.9 (E)				

* Denosumab (60 mg) was administered subcutaneously.

# Hemodialysis was performed.

M: morning; E: evening.

### Patient 3:

A 56-year-old female presented to the emergency department with abdominal pain and recurrent vomiting for one week. Investigations revealed PTH-dependent hypercalcemia ( Table 1 ). Abdominal CT revealed a bulky and edematous pancreas with peripancreatic fat stranding and bilateral tiny, non-obstructing renal calculi. The patient was managed with intravenous normal saline and injectable salmon calcitonin. Her hypercalcemia improved initially but increased again after three days, probably due to calcitonin tachyphylaxis. Acute pancreatitis was managed with nil per oral. Denosumab (60 mg) was subcutaneously administered on day 5 of admission. Her calcium levels decreased to 10.1 mg/dL after two days of denosumab administration ( Table 2 ). Her serum calcium level remained < 10.0 mg/dL until the time of parathyroid surgery, which was performed two weeks after initial injection. Intraoperative PTH decreased by > 75%, and histopathology revealed a single parathyroid adenoma. The postoperative period was unremarkable.

### Patient 4:

A 47-year-old male presented to the emergency department with fever, cough, and breathlessness for one week. Since this was during the coronavirus disease 2019 (COVID-19) pandemic, real-time PCR for SARS-CoV-2 was positive and HRCT chest revealed features of COVID-19 pneumonia. His blood work-up revealed hypercalcemia (serum total calcium, 15.3 mg/dL) and elevated PTH levels (2,308 pg/mL) ( Table 1 ). Ultrasonography of the neck revealed enlargement of all four parathyroid glands. Sestamibi parathyroid scan showed increased uptake in two regions on the right side of the thyroid gland. He was also found to have AKI (serum creatinine, 1.8 mg/dL). He was managed with intravenous fluids, parenteral salmon calcitonin, and denosumab (60 mg, subcutaneously). His calcium levels subsided on the second day of treatment ( Table 2 ) and remained < 11.0 mg/dL until surgery. He underwent bilateral neck exploration and surgical excision of all four glands. The left inferior parathyroid gland was re-implanted into the left forearm. Histopathological examination revealed three parathyroid adenomas and hyperplasia of one gland. The final diagnosis was multiple endocrine neoplasia type 1. He developed hypocalcemia during the postoperative period and required calcium and activated vitamin D (calcitriol) supplements.

## DISCUSSION

Surgery is the mainstay of treatment for PHPT. However, patients who present with severe hypercalcemia must be stabilized medically to attain calcium levels around 12 mg/dL prior to parathyroidectomy to reduce the intraoperative risk associated with high calcium levels ( 3 ).

Patients are started on saline diuresis and parenteral salmon calcitonin, which has a short-term use due to tachyphylaxis, followed by parenteral bisphosphonate while awaiting surgery. For patients who present with AKI, which is mostly the case for severe hypercalcemia, bisphosphonates are not a good option. Hence, in these patients denosumab can be used as a bridge therapy ( 3 - 5 ).

Denosumab is approved for the treatment of osteoporosis in postmenopausal women and men with low bone mineral density, for glucocorticoid-induced osteoporosis with high risk for fracture, as treatment to increase bone mass in men at high risk for fracture receiving androgen-deprivation therapy, and in women at high risk for fracture on adjuvant aromatase inhibitor therapy ( 1 , 6 ). It has been reported that in patients with hypercalcemia of malignancy, the time to response was 9 days and duration of sustained efficacy was up to 104 days ( 5 , 7 ). The adverse effects of denosumab are hypocalcemia in patients with vitamin D deficiency and renal impairment (glomerular filtration rate ≤ 30 mL/min or hemodialysis) ( 5 , 7 ).

We successfully used denosumab in all four patients. It took 24-48 h to normalize serum calcium levels. Serum calcium levels remained < 10.0 mg/dL for the following two weeks, without requiring other calcium-lowering strategies. In cases where denosumab was used to manage hypercalcemia in PHPT, it was administered subcutaneously at a reduced dose of 60 mg. It has been reported that after a single dose, serum calcium levels begin lowering after 12 hours with normalization in three days ( 8 - 12 ). We also used a single dose of denosumab (60 mg), and calcium levels begun lowering after 12-24 hours. In all our cases, serum calcium normalized within 48 hours of denosumab administration, which is a very quick response considering other options. Cinacalcet can also be used, although its onset of action is not sufficiently prompt for life-threatening hypercalcemia.

Intravenous bisphosphonates have been used to control calcium levels in such situations. However, there are issues with the use of bisphosphonates. As most patients with severe hypercalcemia have severe systemic illnesses and renal impairment, clinicians hesitate to administer bisphosphonates. Patient 1 had an altered sensorium, AKI, and suspicion of sepsis; therefore, denosumab was considered a much safer option than intravenous bisphosphonates. Similarly, patients 2 and 3 had acute severe pancreatitis, and patient 4 had COVID-19 pneumonia. In all situations, bisphosphonates have the potential to elicit febrile reactions and render monitoring of the clinical course of the disease difficult. Hence, denosumab is considered to be a better and safer option.

In the literature, there are at least five reports describing the use of denosumab as a bridge to surgery for controlling severe hypercalcemia due to PHPT, when immediate surgery was not feasible ( Table 3 ).

**Table 3:** Summary of case reports of severe hypercalcemia due to PHPT managed with denosumab where immediate surgical management was not feasible

Study, year	Number of patients	Age	sex	Baseline total calcium (mg/dL)	Baseline intact-PTH (pg/mL)	Trend to calcium reduction post denosumab	eGFR (ml/min/1.73m^2^)
Current study, 2021	4	75	F	14.1	1,566	36 hours	27
		35	F	12.5	682		67
		56	F	16.1	1,251		43
		47	M	15.3	2,308		43
Eremkina et al., 2020	10	79	F	14.0	513	3^rd^ day	36
		75	M	17.6	773		28
		44	F	13.2	1,052		44
		30	F	15.6	1,423		124
		25	F	13.6	1,423		139
		65	F	14.4	567		46
		40	F	16.0	1,871		39
		61	M	16.4	1,112		19
		46	F	13.6	2,146		85
		68	F	20.0	345		57
Mamedova et al., 2020	1	16	F	16.7	2,152	12 hours	MHD
Rajan et al., 2019	1	60	M	15.3	1,850	–	21.3
Manandhar et al., 2017	1	81	M	14.6	1,395	3^rd^ day	10.0
Pugliese et al., 2016	2	58	M	2.25 mmol/L iCa	1,286	2^nd^ day	27.0
		55	F	2.35 mmol/L iCa	873		38.6

PTH: parathyroid hormone; eGFR: estimated glomerular filtration rate; MHD: maintenance hemodialysis; iCa: ionized calcium.

We did not encounter any problems with denosumab administration, such as febrile or allergic reactions, nor was it described in any reports published so far. Two of our patients (patients 1 and 4) developed hypocalcemia post-surgery, which was managed with oral calcium and vitamin D supplementation (patient 1) or calcium and calcitriol (patient 4).

In conclusion, denosumab should be considered an effective bridge therapy for definitive surgical management in patients with severe hypercalcemia, especially in the setting of renal impairment and severe systemic illness.
